# Self-administration of augmentation therapy for alpha 1-antitrypsin deficiency

**DOI:** 10.5588/ijtldopen.24.0566

**Published:** 2025-01-01

**Authors:** A. Vianello, S. Lococo, L. Corda, M. Torrazza, G. Zuccarini, P. Baderna, F. Cinetto, E. Bargagli, P. Confalonieri, S. Sanduzzi Zamparelli, L. Bertagna De Marchi, B. Molena, F. Lionello, M. Caminati, G. Guarnieri

**Affiliations:** ^1^ Department of Cardiac Thoracic Vascular Sciences and Public Health, University of Padua, Padua, Italy;; ^2^ Referral Centre for Alpha 1-Antitrypsin Deficiency, ASST Spedali Civili, Brescia, Italy;; ^3^ Department of Internal Medicine, AOU Cagliari, Cagliari, Italy;; ^4^ Respiratory Diseases Unit, Ospedale Santo Spirito, Pescara, Italy;; ^5^ Respiratory Pathophysiology and Ventilation Unit, City Hospital, Saluzzo, Cuneo, Italy;; ^6^ Department of Medicine, University of Padua, Padua, Italy;; ^7^ Respiratory Diseases Unit, Department of Medical and Surgical Sciences & Neurosciences, University of Siena, Siena, Italy;; ^8^ Department of Pulmonology, University Hospital of Cattinara, Trieste, Italy;; ^9^ Department of Clinical Medicine and Surgery, Federico II University, Naples, Italy;; ^10^ Allergy Unit and Asthma Center, Verona University Hospital, Verona, Italy.

**Keywords:** self-medication, emphysema, healthcare professionals, AATD

Dear Editor,

Alpha 1-antitrypsin deficiency (AATD) is a rare, underdiagnosed genetic disorder that results in reduced serum concentrations and/or functionality of alpha 1-antitrypsin (AAT), a serine protease inhibitor. Clinically, patients with AATD have an increased risk of developing emphysema, cirrhosis, panniculitis and vasculitis, as well as a greater susceptibility to viral infection and persistent infections.^1^ Currently, the only condition-specific treatment for AATD-associated diseases is weekly intravenous (IV) augmentation treatment with human AAT (AAT therapy), at a typical dose of 60 mg/kg. This has been proven to effectively delay the progression of emphysema according to computed tomography (CT) measured lung density.^2,3^ However, given the need to frequently access an infusion centre, commitment to AAT therapy may be challenging and adversely impact patients’ independence and/or quality of life (QoL).^4^ Self-administration of IV AAT therapy may therefore be an attractive option that eliminates the need to attend a hospital or outpatient infusion centre.^5,6^ Despite its potential benefits, the demand for self-administration is disappointingly low: a survey conducted in the USA involving individuals participating in the AlphaNet Disease Management and Prevention Program revealed that only 424 out of the 5,266 (8.1%) adult patients surveyed self-administer their AAT infusions.^7^ Given the paucity of data on the prevalence of self-administration by AATD patients, we investigated its use and why patients might decline to participate in self-administration programs in Italy.

A survey based on semi-structured phone interviews with patients currently receiving IV AAT therapy (Respreeza^®^, CSL Behring, King of Prussia, PA, USA; or Prolastin^®^-C Liquid, Grifols Therapeutics, Barcelona, Spain) was conducted between 1 June to 31 December 2023. The participants were identified by lung specialists working at the referral centres accredited by the *Associazione Nazionale Alfa1-AT Onlus* (Italian association of AATD patients). All patients older than 18, diagnosed with severe AATD-related emphysema, and currently receiving IV AAT therapy at any of the referral centres were included in the study. The interview script included questions on the patient’s baseline and treatment-related data. The patients were also asked to rate the overall severity of their respiratory symptoms as severe or non-severe. Patients whose therapy involved a healthcare professional (HCP) were asked if they were willing to consider switching to self-infusion and, if not, why not. The need for ethical approval was waived by the local Ethics Committee of the University of Padua Medical Centre, Padua, Italy (the coordinating centre) as the data collected were considered an integral part of the patient’s clinical history. All participants signed informed consent statements authorising the use of their de-identified clinical data for research, analysis, and reporting purposes (the data were anonymised by assigning a de-identified code to each file).

Overall, 50 subjects were contacted by the lung specialists working at nine referral centres, and 45 agreed to participate in the survey (Table 1). Two respondents (4.4%) were already successfully managed at home via AAT self-administration. Of the remaining 43, 31 regularly attended a healthcare facility for their AAT therapy, and 12 received therapy at home administered by a district nurse. The median treatment duration was 70 months (range 2–336). Of the 43 patients not self-administering, 18 (41.9%) were unaware that the therapy could be self-administered, and 13 (30.2%) stated they were willing to switch from HCP-administered therapy to a self-infusion programme, on condition that they were provided adequate training. The patients were divided into two groups depending on their willingness to enroll in a self-administration program. All patients willing to switch lived in Northern-Central Italy; none were resident in Southern Italy (13/32 vs 0/11; *P* = 0.018; Table 1). Patients willing to switch tended to be recipients of treatment for longer periods than those who were unwilling (84 months, range 2–336 vs 60 months, range 2–252; *P* = 0.141). Reasons for being unwilling to switch are detailed in Table 2. In over 90% of the cases, the perception of lacking the necessary skills was the main obstacle to participating in a self-administration program.

**Table 1.** Baseline demographic and clinical data of patients receiving AAT treatment by healthcare professionals and treatment-related data.

Baseline demographic and clinical data	All cases *n* (%)	Willing to switch *n* (%)	Unwilling to switch *n* (%)	*P*-value*
Subjects, *n*	43	13 (30.2)	30 (69.8)	NA
Age, years, mean ± SD	60.1 ±13.5	57.9 ± 11.6	61.0 ± 14.3	0.503
Sex: M/F	22/21	8/5	14/16	0.370
BMI, kg/m^2^, mean ± SD	23.4 ± 4.5	23.7 ± 5.1	23.3 ± 4.6	0.840
Educational status^†^				0.236
High school diploma or less	3 (21.4)	3 (37.5)	0 (0)	
College classes or college graduate	9 (64.3)	4 (50.0)	5 (83.3)	
Graduate	2 (14.3)	1 (12.5)	1 (16.7)	
Geographical area of residence: Northern-Central/Southern Italy	32/11	13/0	19/11	0.018
Genotype: Pi*ZZ/other^‡^	21/22	6/7	15/15	0.999
Patients with severe respiratory symptoms	23 (53.4)	6 (46.1)	17 (56.7)	0.744
Patients with comorbidities	6 (13.9)	2 (15.4)	4 (13.3)	0.526
AAT treatment-related data				
Treatment duration, months, median (range)	70 (2–336)	84 (2–336)	60 (2–252)	0.141
Infusion setting				
Hospital	30 (71.4)	9 (69.2)	21 (72.4)	0.736
Outpatient infusion clinic	1 (2.4)	0 (0)	1 (3.4)	
Home	12 (27.9)	4 (30.7)	8 (26.6)	

* Refers to differences between ‘Willing to switch’ and ‘Unwilling to switch’ groups.

† Data on educational status were only available for residents in North-East Italy.

‡ Refers to the analysis of mutations of SERPINA1 gene by allele-specific polymerase chain reaction method and/or direct sequencing.

AAT = alpha 1-antitrypsin; SD = standard deviation; M = male; F = female; BMI = body mass index.

**Table 2.** Reasons why patients surveyed were unwilling to switch to self-administration of therapy.

Reasons given	*n* (%)
Lack of skills	25 (78.1)
Fear of making mistakes while preparing the medication	19 (59.3)
Fear of forgetting to self-administer the medication	19 (59.3)
Anxiety and depression linked to the idea of self-administration	18 (56.2)
The loss of regular nurse visits at home	17 (53.1)
Extreme fear of needles	10 (31.2)
Concerns about financial constraints	4 (12.5)
Interference with normal living activities	2 (6.3)
Fear of getting an infection	0 (0)

Although self-administration can reduce the burden of therapy in as many as 30–50% of AATD patients and can be easily learned and safely performed at home,^8^ our study shows that its use is limited in Italy. In line with the results of a previous survey,^7^ only 4.4% of the surveyed patients successfully managed their AAT therapy via self-administration. The most frequently cited reason for declining were that they did not possess the skill or the confidence to self-administer. As lack of knowledge seems to be the patients’ primary consideration, we assume that efforts to increase awareness about the possibility of self-administration, and to train patients to safely and confidently self-administer their infusions, could encourage many to choose this alternative. According to recently published recommendations by an expert consensus, training should focus on all the basics of self-administration.^9^ To note, the survey uncovered that nearly half of the study population was aware that self-administering is an option; most also declared that they would switch to a self-infusion program if training were made available. This finding suggests that efforts should be made to provide HCPs with knowledge about this option so that they can inform, enable and encourage patients to consider this alternative.^10^ To identify factors associated with unwillingness to switch, we found that the geographical area where a patient lived was crucial for this decision. Indeed, all 11 subjects in Southern Italy declined to participate in self-administration programs compared to 19 out of 32 (59.4%) residing in Northern-Central Italy. These regional differences may be due to psychosocial and cultural factors, in particular, weaker family and friend relationships reflecting in social isolation and limiting the availability of a network of non-professional caregivers supporting self-medication.^11^

Our study is limited, given the small number of patients examined. Notably, the number of surveyed patients corresponds to approximately 10% of the estimated number of diagnosed AATD cases in Italy.^12^ Practically all clinical studies examining patients with rare diseases (such as AATD) have similar limitations.^13^

Despite this limitation, the study’s data confirms that healthcare organisations should reinforce efforts to give timely information to all HCPs on the possibility of self-administration of AAT therapy. By providing training and monitoring for patients to self-administer their augmentation therapy, we can improve their treatment satisfaction and QoL.
